# Rapid response of stage IV colorectal cancer with APC/TP53/KRAS mutations to FOLFIRI and Bevacizumab combination chemotherapy: a case report of use of liquid biopsy

**DOI:** 10.1186/s12881-019-0941-5

**Published:** 2020-01-03

**Authors:** Alexander Hendricks, Philip Rosenstiel, Sebastian Hinz, Greta Burmeister, Christoph Röcken, Kathrin Boersch, Clemens Schafmayer, Thomas Becker, Andre Franke, Michael Forster

**Affiliations:** 1 Department of General and Thoracic Surgery, University Hospital Schleswig-Holstein, Campus Kiel, Kiel, Germany; 2 Present Address: Department of General Surgery, University Medicine Rostock, Schillingallee 35, 18507 Rostock, Germany; 3 Department of Pathology, Christian-Albrechts University, Kiel, Germany; 4 Institute of Clinical Molecular Biology, Christian-Albrechts University, Kiel, Germany

**Keywords:** Metastatic colorectal cancer, Circulating tumour DNA, Cell free DNA, Liquid biopsy, Chemotherapy resistance

## Abstract

**Background:**

Liquid biopsies of blood plasma cell free DNA can be used to monitor treatment response and potentially detect mutations that are present in resistant clones in metastatic cancer patients.

**Case presentation:**

In our non-interventional liquid biopsy study, a male patient in his fifties diagnosed with stage IV colorectal cancer and polytope liver metastases rapidly progressed after completing chemotherapy and deceased 8 months after diagnosis. Retrospective cell free DNA testing showed that the *APC/TP53/KRAS* major clone responded quickly after 3 cycles of FOLFIRI + Bevacizumab. Retrospective exome sequencing of pre-chemotherapy and post-chemotherapy tissue samples including metastases confirmed that the *APC/TP53/KRAS* and other major clonal mutations (*GPR50, SLC5A, ZIC3, SF3A1* and others*)* were present in all samples. After the last chemotherapy cycle, CT imaging, CEA and CA19–9 markers validated the cfDNA findings of treatment response. However, 5 weeks later, the tumour had rapidly progressed.

**Conclusion:**

As FOLFIRI+Bevacizumab has recently also been associated with sustained complete remission in a *APC/TP53/KRAS* triple-mutated patient, these driver genes should be tested and monitored in a more in-depth manner in future patients. Patients with metastatic disease should be monitored more closely during and after chemotherapy, ideally using cfDNA.

## Background

Blood plasma “liquid biopsy” from a cancer patient and the analysis of circulating tumour DNA (ctDNA) enables the diversity of the mutational patterns to be monitored over the course of disease at serial timepoints, giving new clinically actionable insights into the therapeutic effectivity. We here report on a case that illustrates how treatment response could be detected early from two blood samples. This case comes from a large ongoing exploratory study whose results were not used for treatment intervention.

In colorectal cancer patients diagnosed with organ metastases, systemic therapy with chemotherapy and targeted antibodies or inhibitors is regularly based on the molecular characterization of the tumour [1]. Usually, molecular testing is based on tissue samples obtained by surgical resection, or on biopsies at time of initial diagnosis. The actual drug response is routinely monitored by imaging methods and tumour markers, but it could be monitored more specifically by serial blood based liquid biopsies.

## Case presentation

**Fig. 1:**
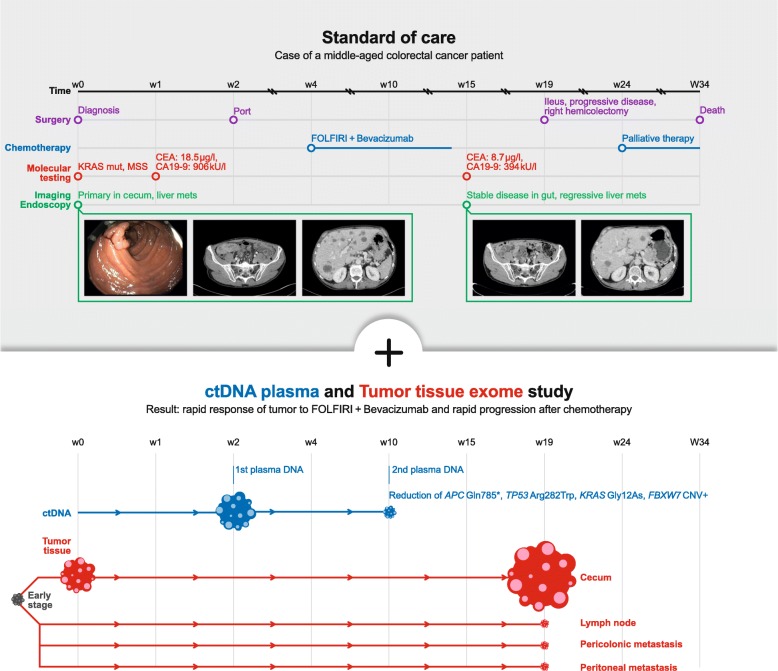
Blood plasma ctDNA and tumour tissue sequencing reveals rapid response to FOLFIRI+Bevacizumab and rapid progression after chemotherapy

We report on a male patient in his fifties of North European ancestry with stage IV colorectal cancer and no known familial history of cancer. The patient had not participated in colon cancer screening tests. Clinical symptoms were unexplained weight loss for a period of six months before diagnosis. Initial diagnosis then presented a primarily metastasized adenocarcinoma of the cecum and bilobular hepatic metastases. Due to polytope bilobular liver metastasis surgical resection was not indicated, and he was admitted to a palliative chemotherapy (CTX). Figure 1 shows the course of events.

The patient gave informed written consent for serial blood sample collection for biomarker analysis and the study was approved by Kiel University’s medical faculty ethics board (#A110/99). For analysis of ctDNA, we obtained a pre-treatment blood sample in week 2 and a mid-treatment blood sample in week 10, after three cycles of chemotherapy. The patient opted out of further blood sampling when he progressed. The blood samples were collected in Streck cfDNA BCT tubes from which plasma was centrifuged according to the manufacturer’s recommendations and stored at − 20 °C until DNA isolation. The plasma was thawed at room temperature. DNA was isolated from the plasma using the PerkinElmer NEXTprep-Mag cfDNA kit according to the manufacturer’s protocol. Ultra-deep sequencing of the ctDNA was performed on Illumina NextSeq 500 with 2x150bp reads using the PANCeq pan-cancer panel [2].

For analysis of tumour tissue mutations, we obtained DNA from a pre-treatment colonoscopy biopsy from the primary tumour in the cecum in week 0 and four tissue samples from an emergency hemicolectomy in week 19 (cecum, lymph node, pericolonic and peritoneal metastases). The tissue samples were obtained from formalin-fixed paraffin embedded samples after HE staining and histological identification and marking of cancer cell regions by the pathological laboratory. Tumour DNA was isolated from the tissue using the RecoverAll™ Total Nucleic Acid Isolation Kit for FFPE (ThermoFisher Scientific Inc) according to the manufacturer’s protocol. Exome sequencing of the tissue DNA samples, and a patient-matched blood buffy coat DNA sample was performed on Illumina NovaSeq 6000 with 2x150bp reads using the IDT xGen Exome Research Panel (Integrated DNA Technologies, Inc.) according to the manufacturers’ protocols. Sequence data analysis was performed with GenSearchNGS (Phenosystems S.A.), Alamut Visual (Interactive Biosoftware), IGV and pibase [3–5].

Initial mutational profiling of a colonoscopy sample from week 0 within the routine diagnostics of the pathological analysis revealed the following: *KRAS* mutation p.G12D; no mutation in exon 15 of BRAF; MSI stable. Our blood sample from week 2 revealed the *KRAS* mutation and four more major tumour mutations in the following genes (see Table 1): *APC* (in 49% of sequences), *TP53* (39%), *KRAS* (32%), *THSD7B* (20%), and a copy number amplification of a chromosomal segment on chromosome 4 containing *FBXW7*. The copy number amplification was deduced from a genomic stretch of germline polymorphisms with near-identical allele frequencies of around 0.25 (see Additional file 1: Table S1) instead of the expected allele frequency of 0.50. Retrospective exome sequencing of the initial cecum tumour tissue biopsy confirmed these mutations and the *FBXW7* amplification and detected further major clonal mutations (Additional file 2: Table S2) that were not covered by the ctDNA sequencing panel.

**Table 1:** Plasma cell free DNA tumour molecular genetic results in HGVS nomenclature, with tumour allele frequency (TAF) and sequence depth at mutation

Gene/Transcript	Genomic alteration (hg19)	Protein alteration	COSMIC or other ID	TAF pre-chemo	Depth	TAF after cycle 3	Depth
*APC* NM_001127511.2	chr5 g.112173590C > T	p.(Gln749*)	COSM 4166473	49%	6171	6%	7055
*TP53* NM_000546.5	chr17 g.7577094G > A	p.(Arg282Trp)	COSM 1636702	39%	4369	5%	4875
*KRAS* NM_033360.2	chr12 g.25398284C > T	p.(Gly12Asp)	COSM521	32%	3910	4%	5003
*THSD7B* NM_001080427.1	chr2 g.137988706G > A	p.(Glu575Lys)	rs746487130	21%	5101	3%	5467
*FBXW7* NM_001013415.1	amplification (CNV)	–	–	ca. 25%^1^	ca. 5000	ca. 43%^1^	ca. 7000

^1^allele frequency of germline polymorphims on a chromosome 4 segment containing *FBXW7*

Based on the mutation in the *KRAS* gene, chemotherapy with FOLFIRI + Bevacizumab (standard dosages) commenced shortly after initial diagnosis. After three cycles of chemotherapy, the blood sample from week 10 revealed major changes in the tumour allele frequencies: *TP53* (6%), *APC* (5.5%), *KRAS* (4%), and *THSD7B* (3%). The amplification of the chromosome segment with *FBXW7* was not clearly detectable any longer in the cfDNA (polymorphism allele frequencies 43–52%). After six cycles of chemotherapy, a re-staging by CT-scan was performed in week 15 and suggested a stable disease of the primary tumour and showed a regression of the liver metastasis (Fig. 1). In parallel, the routinely tested cancer markers CEA and CA19–9 dropped, from 18.5 μg/l to 8.7 μg/l, and from 906kU/l to 394kU/l, respectively.

**Fig. 2:**
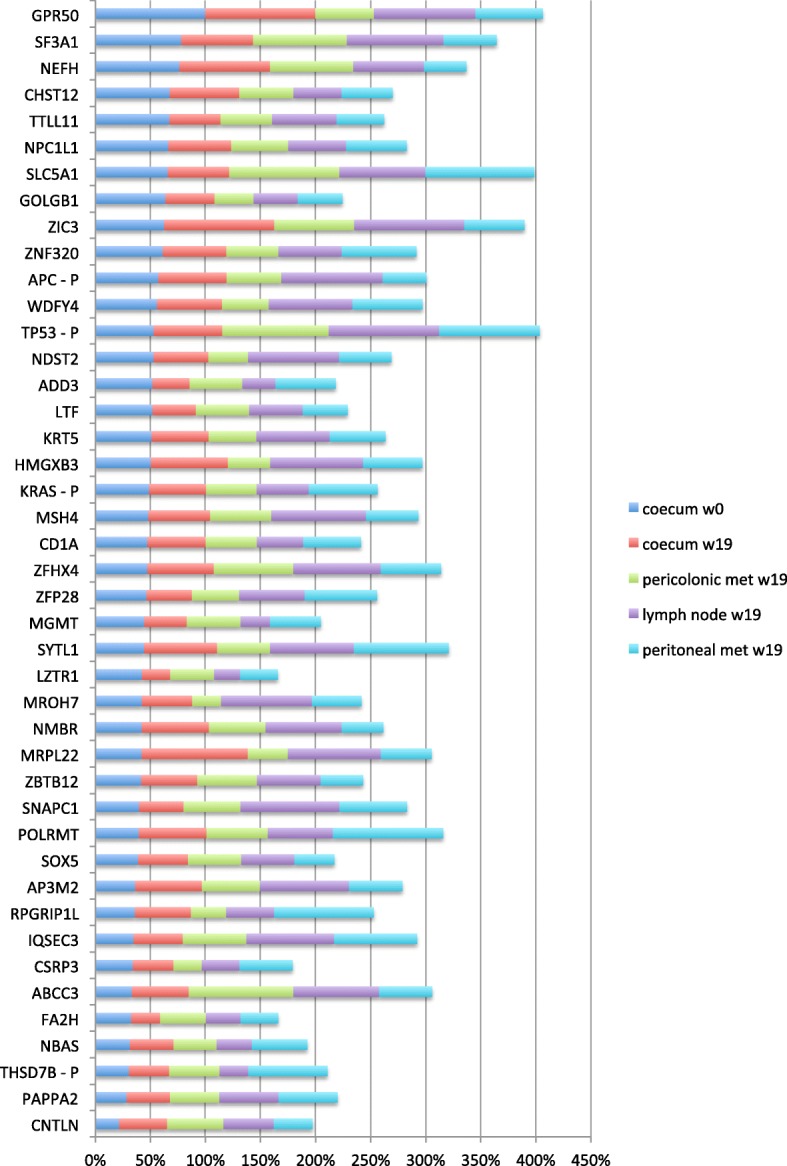
Horizontal stacked bar chart showing normalized somatic mutation allele frequencies detected in tumour tissue samples by whole exome sequencing. The mutation with highest tumour allele frequency in a sample is scaled to 100% and the remaining mutations are scaled accordingly. The chart compares the most prominent tumour mutations between samples, irrespective of the tumour cell content in a tissue sample. P marks genes that were covered in plasma cfDNA pan-cancer sequencing

However, four weeks after the CT scan the patient was admitted to the emergency room with an ileus caused by a substantial increase in size of the primary tumour, a circumferentially growing tumour of 4 × 3.5 cm size at the ileocecal valve. In emergency surgery a right hemicolectomy was performed. Retrospective exome sequencing of the tissue samples obtained from the hemicolectomy (cecum, lymph node, pericolonic and peritoneal metastases) confirmed that the FBXW7 amplification was no longer clearly detectable in the cecum, lymph node, or peritoneal metastasis, but possibly in the pericolonic metastasis sample. The major clonal mutations detected in the pre-treatment biopsy remained conserved in all post-treatment samples, with no new major clonal mutations detected (Fig. 2 and Additional file 2: Table S2).

After surgery, a second line chemotherapy with FOLFOX + Bevacizumab was started. Unfortunately, short-term imaging of the tumour burden exposed progressive disease, so that shortly afterwards a 3rd line chemotherapy by Trifluridin + Tipiracil was started. Therapy monitoring by ultrasound imaging of the hepatic metastasis presented substantial progression of the tumour load and hence inefficiency of the systemic therapy. The patient was transferred to best supportive care and deceased 8 months after initial diagnosis.

## Discussion and conclusion

We here illustrate the potential clinical use and benefit of serial liquid biopsies. Our middle-aged male patient was diagnosed with a metastasized colorectal carcinoma and progressed rapidly over the course of eight months from initial diagnosis to decease.

The initial ctDNA analysis prior to the first cycle of chemotherapy was concordant to the routine pathological mutational profiling in terms of mutational patterns. The different levels of tumour allele frequencies in the blood plasma cell free DNA suggest that the tumour was heterogeneous with different tumour clones. The first mutation may have occurred in *APC*, followed by *TP53*, *KRAS*, amplification of *FBXW7*, and mutation in *THSD7B*. Truncating *APC* mutations are colorectal cancer initiating mutations that occur together with *TP53* mutations and *KRAS* mutations in 20% of stage IV colorectal cancers [6].

At time of diagnosis, polytope hepatic metastases were present. Consequently, and compliant with recent guidelines chemotherapy with FOLFIRI + Bevacizumab was started. Initially the systemic treatment had a positive effect on the tumour burden. In the course of chemotherapy, we obtained another liquid biopsy which indisputably showed a significant change in the mutated genetic pattern. Halfway through treatment, the dominant *APC/TP53/KRAS*-mutated clone was nearly eradicated due to treatment with FOLFIRI + Bevacizumab. In parallel, the *FBXW7* amplification was eradicated or nearly eradicated. Complete tumour response after treatment with FOLFIRI + Bevacizumab is rare but there is a recent report in the literature on the complete remission of a APC/TP53/KRAS triple-mutated stage IV colorectal cancer patient for over 10 years [7]. The response seen in our patient’s CT scan at week 15 (Fig. 1) suggests that the eradicated liver metastases may have harboured predominantly cells from the triple-mutation tumour clone. The spatial heterogeneity of CNVs and homogeneity of point mutations that we detected in our patient ties in with previous reports of colonic cancer [8].

We suggest that metastatic patients should routinely be offered liquid biopsy testing with frequent blood sampling, and that all of the major driver genes are covered. As seen in our patient, liquid biopsy can detect drug response to a treatment, it may detect progression early, and, after remission, it may also be used for the early detection of disease recurrence, as in the current IMPROVE-trial (NCT 03637686).

## Supplementary information

**Additional file 1 Table S1.** Allele frequencies of germline polymorphims on chromosome 4, showing copy number amplifications in the pre-chemo plasma cfDNA.

**Additional file 2 Table S2.** Somatic mutations detected using exome sequencing of cancer tissue samples versus blood bufy coat normal DNA.

## Data Availability

The datasets generated and analysed during the current study are available in the European Genome-phenome Archive (EGA) repository under the study accession ID EGAS00001004088.

## Abbreviations

CA19–-9: Carbohydrate antigen 19–-9
CEA: Carcinoembryonic antigen
cfDNA: Cell free DNA
CT: Computer tomography
ctDNA: Circulating tumour DNA
CTX: Chemotherapy
MSI: Microsatellite instability
VEGF: Vascular endothelial growth factor
